# Reconstruction of kidney renal clear cell carcinoma evolution across pathological stages

**DOI:** 10.1038/s41598-018-20321-4

**Published:** 2018-02-20

**Authors:** Shichao Pang, Yidi Sun, Leilei Wu, Liguang Yang, Yi-Lei Zhao, Zhen Wang, Yixue Li

**Affiliations:** 1 Department of Statistics, School of Mathematical Sciences, Shanghai Jiao Tong University, Shanghai, 200240 China; 2 Department of Bioinformatics and Biostatistics, MOE LSB and LSC, State Key Laboratory of Microbial Metabolism, Joint International Research Laboratory of Metabolic & Developmental Sciences, School of Life Sciences and Biotechnology, Shanghai Jiao Tong University, Shanghai, 200240 China; 3 Key Lab of Computational Biology, CAS-MPG Partner Institute for Computational Biology, Shanghai Institutes for Biological Sciences, Chinese Academy of Sciences, Shanghai, P.R. China; 4 CAS Key Laboratory of Systems Biology, CAS Center for Excellence in Molecular Cell Science, Institute of Biochemistry and Cell Biology, Shanghai Institutes for Biological Sciences, Chinese Academy of Sciences, 320 YueYang Road, Shanghai, 200031 China; 5 University of Chinese Academy of Sciences, Shanghai, 200031 China; 6 Shanghai Center for Bioinformation Technology, Shanghai Industrial Technology Institute, Shanghai, P.R. China; 7 Collaborative Innovation Center for Genetics and Development, Fudan University, Shanghai, P.R. China

## Abstract

Although numerous studies on kidney renal clear cell carcinoma (KIRC) were carried out, the dynamic process of tumor formation was not clear yet. Inadequate attention was paid on the evolutionary paths among somatic mutations and their clinical implications. As the tumor initiation and evolution of KIRC were primarily associated with SNVs, we reconstructed an evolutionary process of KIRC using cross-sectional SNVs in different pathological stages. KIRC driver genes appeared early in the evolutionary tree, and the genes with moderate mutation frequency showed a pattern of stage-by-stage expansion. Although the individual gene mutations were not necessarily associated with survival outcome, the evolutionary paths such as VHL-PBRM1 and FMN2-PCLO could indicate stage-specific prognosis. Our results suggested that, besides mutation frequency, the evolutionary relationship among the mutated genes could facilitate to identify novel drivers and biomarkers for clinical utility.

## Introduction

Kidney cancer, also called renal cell carcinoma (RCC), is one of the most common cancers in both men and women. It was estimated that 63,990 new cases and 14,400 deaths (including 9,470 men and 4,940 women) of RCC would likely occur in 2017^1^. According to pathological features and auxiliary characters such as particular driver gene or responses to therapy, RCC was divided into three major subtypes^2^; among them clear cell renal carcinoma (ccRcc) is the most common subtype, accounting for 65–75% of all RCC^3^. Genomic studies have identified several genes, i.e., VHL (von-Hippel Lindau tumor suppressor), PBRM1 (polybromo 1), BAP1 (BRCA1-associated protein-1) and SETD2 (SET domain containing2), as driver genes for RCC. Genetic mutations in these driver genes are able to regulate hypoxia inducible factor α subunits (such as HIF-1α and HIF-2α), leading to the activation of hypoxia pathways in RCC^4^. Among these genes, only the mutation of BAP1 showed significant correlation with poor survival^5^. Some researchers investigated mutation frequency differences of driver genes between early and late stages and found that PBRM1 or BAP1 mutation took place more often in late stages(III&IV)^6^. But the detailed dynamics of these somatic mutations during KIRC progression were not clarified yet.

It has been recognized that cancer is a disease of clonal evolution in body^7^, and the evolutionary mechanism can illuminate its progression^8^. As an example, the accumulations of genetic mutations have a significant impact on tumor progression, and cell diversities ended up in tumor heterogeneity^9^. The clones possess different fitness to survival and proliferation, and if the proliferative speed is fast enough, the survival status of tumor cells doesn’t matter anymore^10^. So the evolutionary path of the clone with highest fitness also represents the most efficient proliferation. Based on genome-wide variations derived from next-generation sequencing, diverse methods were proposed to construct tumor evolution^11^.

In the current case, we reconstructed a KIRC evolution process based on the cross-sectional data from The Cancer Genome Atlas (TCGA). Although great challenges exist in the computational reconstruction of tumor evolution for CNVs, KIRC can be well exempt from the challenges because its tumor initiation and evolution are predominated by somatic single nucleotide variations (SNVs) compared to other cancers^12^. Especially, we associated the evolution of KIRC with pathological stages and found that the pathology of KIRC fitted well to the reconstructed phylogenetic tree in a fashion of stage-by-stage expansion. In addition, despite a poor prognostic biomarker  for VHL mutation itself ^13^, we found the evolutionary path between VHL and PBRM1 varied across stages, which would be an effective indicator of prognosis.

## Results

### Mutational landscape of Kidney renal clear cell carcinoma from TCGA cohort

**Figure 1:**
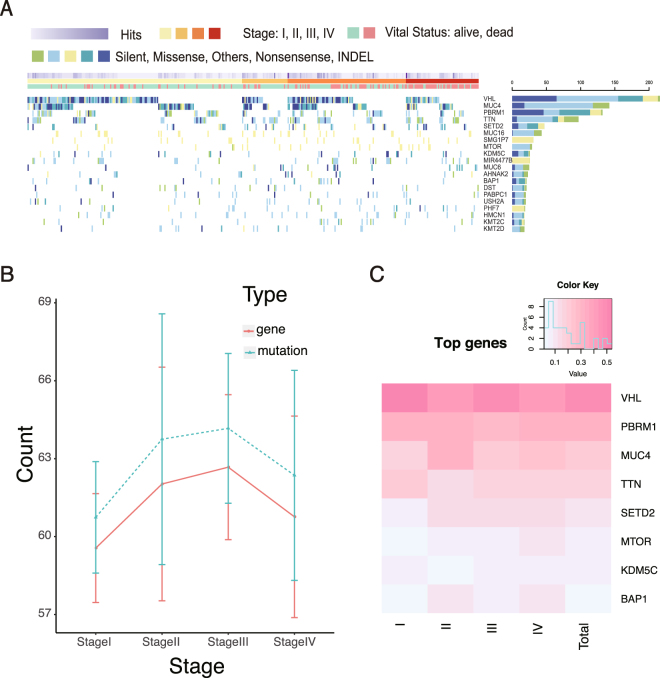
Overall features of KIRC mutation. (**A**) Distribution of the genes with high mutation frequency. (**B**) Mutation frequency and gene kinds per patient in different pathological stages. (**C**) Heat map of stage-specific frequency for the top 8 genes with high mutation frequency.

Among 499 primary KIRC specimens in TCGA, only 417 samples have clear information of pathological stages (Fig. 1A and Supplementary Table 1). After filtering hyper-mutated samples, we involved the somatic mutations with oncotator annotations in UCSC dataset. As a result, KIRC driver genes (i.e., VHL, PBRM1, SETD2 and BAP1) showed the topmost mutation frequency. The overall mutation frequency among different pathological stages (Fisher’s exact test p-value = 0.5546) and the number of mutated genes (Fisher’s exact p-value = 0.5751) exhibited no significant difference (as shown in Fig. 1B). However, the mutation frequency of BAP1 showed a significant increase among different pathological stages (logistic regression p-value = 0.0062, Supplementary Fig. 1A) which was consistent with the previous reports, but no significant trend was observed for PBRM1 (Supplementary Table 2). VHL had a degressive tendency in the mutation frequency (logistic regression p-value = 0.028, Supplementary Fig. 1B), but no stage specificity was observed (Fig. 1C). These findings indicated that most driver genes of KIRC were established at the early pathological stage and became impactive during the tumor progression. Further survival analysis showed that MUC4 mutations were in strong correlation with poor survival in all KIRC samples (log-rank test p-value = 0.018), and in stages I and III samples (log-rank test p-value = 0.0482 and 0.0277, respectively). VHL mutations were found to correlate with poor survival only in stage II (log-rank test p-value = 0.012). Besides, the other genes with high mutation frequency had no correlation with survival outcomes in both overall samples and different pathological stages (Supplementary Table 3).

### Evolutionary reconstruction of Kidney cancer

**Figure 2:**
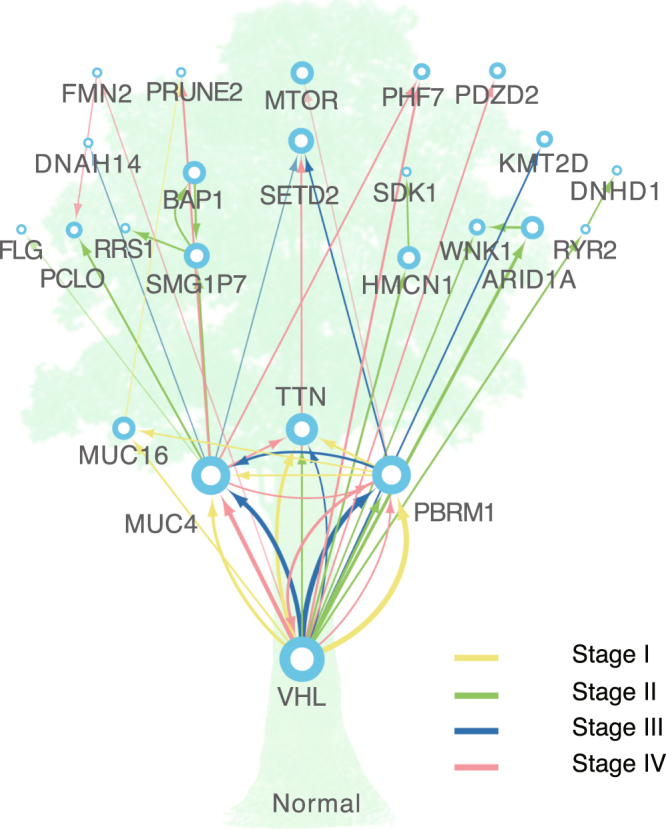
KIRC evolutionary path. This is a consensus graph based on evolutionary trees of each pathological stage. Line width represents the edge weights. Nodes are ordered by pathological stages.

As genetic studies only used the high-frequency mutations in all the samples to identify driver genes, the information for the mutations with moderate frequency in tumor progression and their evolutionary relationships were always missing. To address this point, we reconstructed a KIRC evolutionary path based on mutated genes by Bayesian Mutation Landscape (BML)^14^. Samples  at different stages were  combined and separated in the evolution analysis, which were then integrated to generate a consensus graph. Considering the statistical confidence, we only kept trunk genes of the evolution tree in the graph. As a result, the most probable paths of gene mutations was shown in a tree model. (Fig. 2, see Methods). Additionally, we also incorporated pathological stage information into the KIRC evolutionary tree mentioned above. In the tree model, the genes with both high and moderate mutation frequency were included, as long as they significantly impact on evolutionary efficiency (that is,  mutation of these gene would promote the probability of subsequent gene mutations along evolutionary path). Although not all of the high-impactive genes are  tumor drive genes, their mutations could be sort-of  intermediate in the tumor evolution process.

As shown in Fig. 2, the genes with high mutation frequency were located on the bottom of the trunk in the evolutionary tree, having direct relations with normal nodes. The KIRC deriver genes (VHL, PBRM1 and MUC4) possess  a comparatively high out-degree (defined as the  number of arcs leading away from the node, Supplementary Table 4). The evolutionary paths of VHL, PBRM1, and MUC4 were identified in all pathological stages, indicating that the driver genes played an early and fundamental role in KIRC progression. Although TTN was also one of the genes  located at the bottom of the trunk, its low out-degree and less subsequent mutations limited its roles in KIRC progression. This observation is consistent with the fact that TTN tends to be a passenger gene rather than a driver gene  in cancerology^15^.

The genes with a moderate mutation frequency (i.e., PCLO and WNK1) were also found in the trunk of the evolutionary tree. This was mainly due to their intermediary roles for up- and down-stream genes in the evolutionary path and thereby  significant correlations with the  pathological stages. For instance, WNK1 mutations were found to be enriched in stage II (mutation frequency = 10%), which differed from other stages (Fisher's exact test  p-value = 0.003,  0.004, and   0.017  compared to those  in stages I,  III,  and  IV,  respectively;). The patients with WNK1 mutations in stage II showed a tendency to more gene mutations (Wilcox rank sum test p-value = 0.041). Besides  the correlations with pathological stages, at least one-third of the trunk genes were related to stage-specific survival outcomes (Supplementary Table 5).

Geneontological analysis indicated that the trunk genes involved in ion binding and lipidrelated biological  processes (Supplementary Fig. 2). BML analysis showed that the genes with moderate mutation frequencies had an evolutionary pattern with stage-by-stage expansion (Supplementary Fig. 3). In stage I, the genes with a high mutation frequency (i.e., VHL and PBRM1) were directly connected to normal nodes. In stage II, the trunk genes showed a comparatively high average degree (Supplementary Table 6) in PPI (Protein-Protein Interaction) network, giving an indication that these genes had significant connections to abundance genes and more follow-up variations in later stages. This finding was further supported by the fact that entropy of edges and nodes with high bootstrap score both increased over time (Supplementary Fig. 4), which turned out to be a stage by stage expansion.

### Survival analysis of evolutionary paths

**Figure 3:**
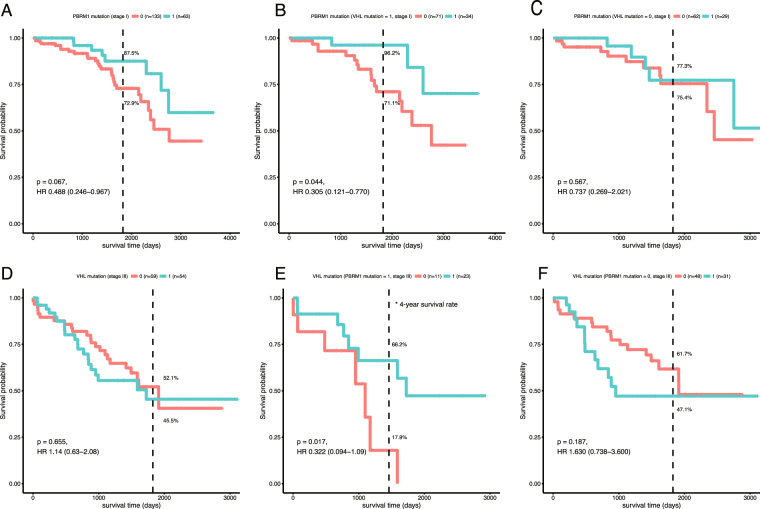
Stage specific survival analysis. (**A**) Survival curve for PBRM1 mutations in stage I. (**B**) Survival curve for PBRM1 mutations with VHL mutations in stage I. (**C**) Survival curve for PBRM1 mutations without VHL mutations in stage I. (**D**) Survival curve for VHL mutations in stage III. (**E**) Survival curve for VHL mutations with PBRM1 mutations in stage III. (**F**) Survival curve for VHL mutations without PBRM1 mutations in stage III. Dash line represents the time point of 5 years.

**Figure 4:**
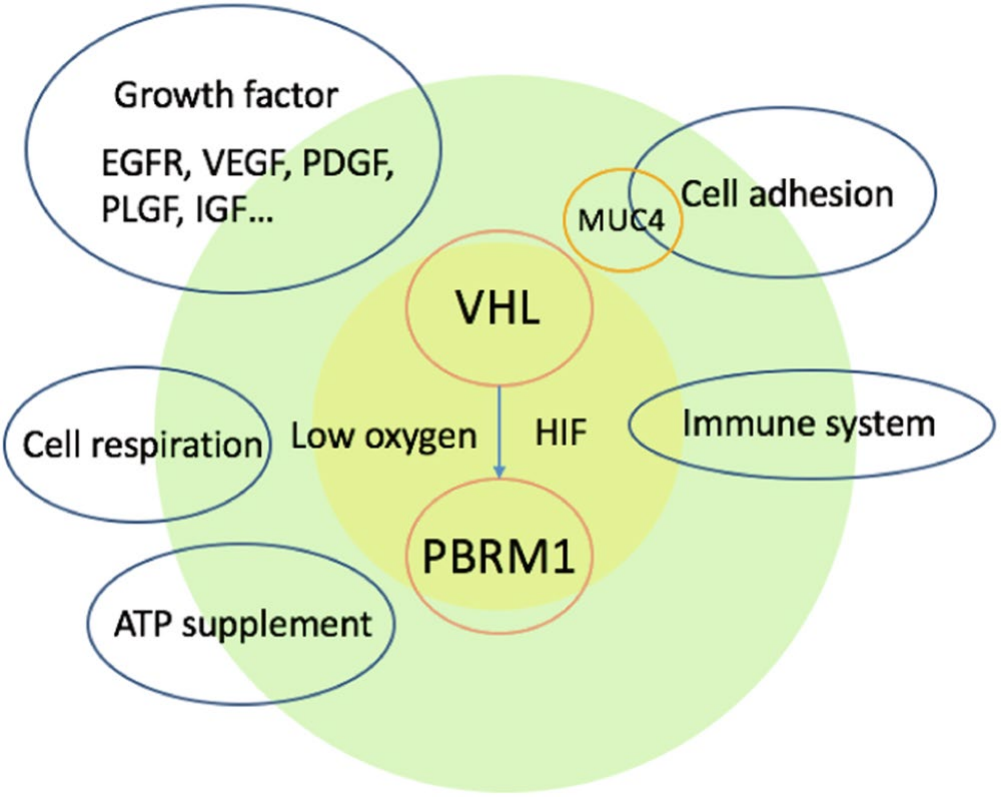
Schematic diagram of biology process during KIRC evolution.

In addition to the mutations of a single gene, the edges between the trunk genes represented their evolutionary relationships in stage progression. Thus, we selected highly weighted edges in different stages, and analyzed the corresponding gene patterns. As a result, VHL and PBRM1 were picked up as the topmost gene pattern with high mutation frequency and close interrelation (VHL-to-PBRM1 path in stage I, and PBRM1-to-VHLpath in stage III). Although no significant association between gene mutations of either VHL or PBRM1 and overall survival was detected, the clinical outcomes of PBRM1 mutations in stage I showed a significant dependence on VHL mutations. In the existence of VHL mutations, the patients with PBRM1 mutations showed a better survival outcome than the ones without PBRM1 mutations (Fig. 3A and B). However, the survival outcome for PBRM1 mutations without VHL mutations could not be distinguished (Fig. 3C). Oppositely, the survival outcome of VHL mutations exhibited a significant dependence on PBRM1 mutations in stage III that existence of PBRM1 mutation resulted in a better survival outcome (Fig. 3D–F). VHL helps an immune system^16^ related E3 ligase to label hypoxia-inducible factor (HIF) 1α and 2α by ubiquitin for degradation^17^. PBRM1 is a co-activator to induce HIF target genes. In tumor cells, anaerobic environment can affect HIF activities, regulating T cell differentiation^18^. However, T cell differentiation is considered as a key factor of tumor immune evasion mechanism in KIRC^19^. The mutation accumulation in trunk genes, especially VHL and MUC4, can regulate cell adhesion^20,21^, helping tumor cells to escape from the immune system. The mutations in PBRM1 can disturb ATP supplement, making cells to adopt anaerobic respiration^22^. As a result, glucoses were overly accumulated, facilitating the vicious cycle of ATP deficit and low oxygen (Fig. 4). Besides, VHL was connected to lots of growth factor^23^, mutations on them also aroused and well-known genes such as  BAP1 and SETD2 raised up frequency later. Reduction of Immune system accompanied with adding confusion of tumor system both increased trunk gene types and amounts in late stages. It is suggested that this stage by stage extension appeared in KIRC mainly due to tumor evasion system.

Another notable evolutionary path was detected between FMN2 and PCLO. The mutation frequency of PCLO was below 5%, and had no significant association with overall survival. PCLO was regarded as a trunk gene in stage II, but its mutations were irrelevant to poor survival in this stage. Together with FMN2, PCLO formed a positive feedback in the evolutionary tree in stage IV, suggesting their roles in accelerating cancer evolution. In stage IV, PCLO mutations were significantly associated with poor survival (log-rank test p-value = 0.0002). Furthermore, the patients with both FMN2 and PCLO mutations had worse survival in stage IV (log-rank test p-value = 1.47e-05) than those with individual gene mutations. This result suggested that the evolutionary paths of FMN2-PCLO would significantly affect the clinical outcomes. Considering the relatively low mutation frequency of both FMN2 and PCLO mutations, we also studied their combinations for prognosis on the expression level. In FMN2 low expressed group, patients with lower PCLO expression had worse survival outcome (separated by median, p-value = 0.0292, Supplementary Fig. 5), which was in agreement with the combination of their mutations.

## Discussion

Cancer evolution models varied these years from simple linear theory^24^, nonlinear or branching theory^25^ to big bang theory^26^ and neutral evolution theory^27^. They shared something in common and had differences as well. Novell proposed the theory of clonal evolution of tumor^28^, and many genomic studies showed the existences of subclones in a tumor^29^. In this theory, the accumulation of mutations would drive early slow-growing subclones into fast-growing subclones, which accelerated tumor progression^30^. Based on this idea, we hypothesized that there was a probable evolutionary path of gene mutations which could drive cancer progression across pathological stages. Although the mutations in the TCGA data tended to happen during tumor initiation before pathological transformation due to the low purity and moderate sequencing depth^31^, mutations occurring in later stages could be detected if they were associated with fast-growing subclones.

As a SNV-dominated cancer, we reconstructed the evolutionary process for KIRC combined with pathological stages by the BML method. Most of the well-known driver genes with high mutation frequency were established before the early stage, but many genes with moderate mutation frequency emerged with a stage-by-stage expansion. One-third of the genes with moderate mutation frequency were associated with the survival outcome, indicating they were not random but involved in the tumor progression of KIRC. Particularly, some gene mutations such as BAP1 had malignant potential before stage progression, but its mutation frequency raised up with stage and had more serious effects in later stages^32^. Besides, topological features of the tree graph model, such as in-degree and out-degree suggested a new point of view to evaluate driver genes in different stages.

Although individual mutated genes were commonly used for prognosis, their validity in KIRC was limited, even if for those highly mutated genes. Our results, however, suggested that the evolutionary relationship between the mutated genes could sometimes be more effective for prognosis. One intriguing example was VHL-PBRM1. We implied that a compensation equilibrium existed between PBRM1 and VHL in the evolution process. In the early stage of KIRC, PBRM1 mutation relieved this process to maintain cellular fitness despite high levels of genomic instability. While in late-stage patients with the PBRM1 mutation had better survival outcome under VHL mutation condition. They might influence each other by regulating HIF activation. Researchers have proved that PBRM1 restrained VHL loss in KIRC^33^, the opposite arrow from PBRM1 to VHL is also worth pondering. Another evolution path mentioned above was from FNM2 to PCLO in stage IV. PCLO gene encoded protein is part of the presynaptic cytoskeletal matrix which is involved in establishing active synaptic zones and in synaptic vesicle trafficking. While FMN2 is a member of the formin homology protein family and plays important roles in the organization of the actin cytoskeleton and in cell polarity. Lots of cytoskeleton or cytoskeletal matrix-related genes were reported functioned with circRNA, so did PCLO and FMN2^34^. On account of their moderate mutation frequency in KIRC, only a few studies on them  were reported. More attention on these moderate mutated genes in specific stages might bring new discoveries.

## Methods

### Data processing

Both genetic and clinical data for 417 KIRC samples were obtained from TCGA Data Portal Bulk Download (http://tcga-data.nci.nih.gov/tcga)^35^, with  a declaration that all TCGA data are now available without restrictions on their use in publications or presentations. Single nucleotide variants (SNV) for these KIRC samples were subsequently annotated by Oncotator^36^ in UCSC cancer browser (UCSC Xena now). After removing hyper mutated samples, we transformed them to a 0/1 matrix (patient x mutation gene) and filtered low mutation frequency (<3) genes in order to lessen bias. Statistical test in Fig. 1B and C were carried out using Fisher’s exact test and Kruskal-Wallis rank sum test.

### Reconstruction of cancer evolutionary process

Bayesian mutation landscape (BML)^14^ is a probability network to reconstruct ancestral genotypes and the paths of mutation accumulation. Since this method requires more samples than gene mutations, we need to reduce gene number for input. G(i) represented the number of genes with mutation frequency larger than i. In order to make gene number approach to sample size N, we adjusted the threshold i of G(i) which satisfied (1) G(i) ≥ N and (2) G(i + 1) < *N*. We used GeneOverlap package^30^ in R to evaluate the overlap degree between two evolution maps of G(i) and G(i + 1) using sample size 30, 60 and 100. We generated 10 times random sampling for each sample size and all of them had a p-values less than 0.05 which means significant similarity. In order to make use of more priori knowledge, we analyzed mutation data by pathological stages (Supplementary Fig. 3). Although the best way to reduce tumor heterogeneity is to use mutation data in different stages of same patient, BML aiming at an efficient data structure to recapitulate the likely sequence of somatic mutation, there is no need to imply hierarchical order of mutations. So we can assume different patients in different stages share the same evolution trunk. Since the data size was stage unequal, we randomly selected 30 samples (with replacement) in each stage for 100 times and built their evolution DAG (Directed Acyclic Graph) using BML algorithm. For stage t, there were Q_t_ edges appeared in the DAG after 100 times bootstrap, and the occurrence frequency for edge i (N_it_) were assigned as its weight, top 1% in each stage was defined as main branch. All the edges with weight larger than 3 were listed in Supplementary Table 7. We constructed the whole process DAG by raw data and annotated stage information from bootstrap result. Some high weight (>10) stage-specific edges lost in raw data DAG were also added. Then we built a network by Cytoscape (version 3.4.0) and adjusted its structure by stage order. We defined the genes appeared in the final evolutionary map as trunk genes. Entropy were counted based on both edge and node weights. For edges’ entropy, their occurrence frequency was calculated as:

1 $${f}_{it}=\frac{{N}_{it}}{100}\,\forall i\in {Q}_{t}$$

f_it_ could also be regarded as edge occurrence time in a single experiment. The probability of f_it_ was calculated as:

2 $$\,{p}_{it}=\frac{{f}_{it}}{{\sum }_{i\in {Q}_{t}}{f}_{it}}$$

We used different weight threshold j to evaluate the entropy of the top edges in each stages,

3 $${E}_{jt}=-\sum _{i < j,i\in {Q}_{t}}{p}_{it}\,\mathrm{log}\,{p}_{it}$$

where j threshold occupied percentage were counted as:

4 $${P}_{jt}=\frac{{E}_{jt}}{{E}_{t}}$$

It is noteworthy that we merged stage 1 and stage 2 together since we were inclined to observe the differences between early and late stages.

5 $${f}_{i(1+2)}=\frac{{f}_{i1}+{f}_{i2}}{2}$$

### Survival analysis and function enrichment

Survival time used in this paper was the time to death or censor event (patients still alive or lost follow-up at the end of the study). Single factor and multifactor survival analyses were performed using log-rank method^37^ and cox model with Breslow^38^ score, respectively. Survival curve was generated by Kaplan-Meier estimator and plotted by R package “survminer”^39^. WEB-based GEne SeT AnaLysis Toolkit^40,41^ were used for function enrichment with parameters set as Bonferroni, p < 0.05.

## Electronic supplementary material

Supplementary Information
