# Supplementary material for: Reconstruction of kidney renal clear cell carcinoma evolution across pathological stages
Source: Sci Rep. 2018 Feb 20;8:3339. doi: 10.1038/s41598-018-20321-4 (PMC5820260; doi:10.1038/s41598-018-20321-4)

Reconstruction of kidney renal clear cell carcinoma evolution across pathological stages

Shichao Pang[1], Yidi Sun[3,4,5], Leilei Wu[2], Liguang Yang[3,5], Yi-Lei Zhao[2*], Zhen Wang[3*], Yixue Li[2,3,4,5,6,7*],

1 Department of Statistics, School of Mathematical Sciences, Shanghai Jiao Tong University, Shanghai, 200240, China;

2 Department of Bioinformatics and Biostatistics, School of Life Sciences and Biotechnology, Shanghai Jiao Tong University, Shanghai, 200240, China;

3 Key Lab of Computational Biology, CAS-MPG Partner Institute for Computational Biology, Shanghai Institutes for Biological Sciences, Chinese Academy of Sciences, Shanghai, P. R. China

4 CAS Key Laboratory of Systems Biology, CAS Center for Excellence in Molecular Cell Science, Institute of Biochemistry and Cell Biology, Shanghai Institutes for Biological Sciences, Chinese Academy of Sciences, 320 YueYang Road, Shanghai, 200031, China

5 University of Chinese Academy of Sciences, Shanghai 200031, China;

6 Shanghai Center for Bioinformation Technology, Shanghai Industrial Technology Institute, Shanghai, P. R. China

7 Collaborative Innovation Center for Genetics and Development, Fudan University, Shanghai, P. R. China

* Correspondence and requests for materials should be addressed to Y.X.L. (email: yxli@sibs.ac.cn), Z.W. (email: zwang01@sibs.ac.cn) or Y.-L.Z. (email: yileizhao@sjtu.edu.cn)

Supplementary Table 1: KIRC sample size of separate stages

Stage	Stage I	Stage II	Stage III	Stage IV	Total
Patients	196	41	113	67	417

Supplementary Table 2: Likelihood test of SMGs distribution in separate stages (Logistic regression)

Gene	p-value(Likelihood)
**PBRM1**	0.3204
**BAP1**	0.0062
**VHL**	0.0285
**SETD2**	0.0691
**TTN**	0.1376
**MUC4**	0.0793
**KDM5C**	0.9838
**MTOR**	0.2877

Supplementary Table 3: Survival outcome of known driver genes

Gene	Stage	p-value(log-rank)
**VHL**	1	0.827
**PBRM1**	1	0.114
**MUC4**	1	0.0482
**TTN**	1	0.893
**SETD2**	1	0.484
**MTOR**	1	0.517
**KDM5C**	1	0.103
**BAP1**	1	0.281
**VHL**	2	0.012
**PBRM1**	2	0.371
**MUC4**	2	0.161
**TTN**	2	0.511
**SETD2**	2	0.0851
**MTOR**	2	0.585
**KDM5C**	2	0.0975
**BAP1**	2	0.807
**VHL**	3	0.655
**PBRM1**	3	0.317
**MUC4**	3	0.0277
**TTN**	3	0.396
**SETD2**	3	0.49
**MTOR**	3	0.899

**KDM5C**	3	0.144
**BAP1**	3	0.345
**VHL**	4	0.914
**PBRM1**	4	0.265
**MUC4**	4	0.372
**TTN**	4	0.215
**SETD2**	4	0.251
**MTOR**	4	0.827
**KDM5C**	4	0.909
**BAP1**	4	0.196
**VHL**	all	0.918
**PBRM1**	all	0.373
**MUC4**	all	0.0179
**TTN**	all	0.945
**SETD2**	all	0.271
**MTOR**	all	0.316
**KDM5C**	all	0.0586
**BAP1**	all	0.101

Supplementary Table 4: Degree of KIRC evolution DAG

Gene	In-degree	Out-degree	In-degree(weighted)	Out-degree(weighted)
**ARID1A**	1	1	31	20
**BAP1**	1	1	13	15
**DNAH14**	1	0	4	0
**DNHD1**	1	0	10	0
**FLG**	1	0	5	0
**HMCN1**	1	1	18	10
**KMT2D**	1	0	11	0
**MTOR**	1	0	4	0
**MUC16**	2	1	22	5
**MUC4**	5	9	155	101
**PBRM1**	5	7	183	86
**PCLO**	2	0	20	0
**PDZD2**	1	0	10	0
**PHF7**	2	0	30	0
**PRUNE2**	2	0	19	0
**RRS1**	1	0	13	0
**RYR2**	1	1	14	10
**SDK1**	1	0	10	0
**SETD2**	3	0	25	0
**SMG1P7**	2	2	36	26

**TTN**	5	1	92	7
**VHL**	1	19	10	485
**WNK1**	2	0	30	0
**FMN2**	0	1	0	5

Supplementary Table 5: Survival outcome of trunk genes

Gene	Stage	p-value(log-rank)
**ARID1A**	1	0.956
**WNK1**	1	1.26E-11
**DNAH14**	1	0.609
**DNHD1**	1	0.758
**FLG**	1	0.443
**FMN2**	1	0.145
**PRUNE2**	1	0.867
**RRS1**	1	0.781
**RYR2**	1	0.611
**SDK1**	1	0.262
**PCLO**	1	0.304
**PDZD2**	1	0.684
**HMCN1**	1	0.0596
**KMT2D**	1	0.855
**MUC16**	1	0.192
**PHF7**	1	0.671
**SMG1P7**	1	0.973
**ARID1A**	2	0.845
**WNK1**	2	0.382
**DNAH14**	2	0.629
**DNHD1**	2	0.000404
**FLG**	2	0.409
**RRS1**	2	0.25
**RYR2**	2	6.95E-07
**SDK1**	2	0.343
**PCLO**	2	0.402
**HMCN1**	2	0.929
**PHF7**	2	0.487
**SMG1P7**	2	0.982
**ARID1A**	3	0.261
**DNAH14**	3	0.738
**DNHD1**	3	0.298
**FLG**	3	0.539
**FMN2**	3	0.451
**RYR2**	3	0.696

**SDK1**	3	0.808
**PCLO**	3	0.214
**PDZD2**	3	0.24
**HMCN1**	3	0.0398
**KMT2D**	3	0.264
**MUC16**	3	0.043
**PHF7**	3	0.457
**SMG1P7**	3	0.254
**ARID1A**	4	0.856
**DNAH14**	4	0.903
**DNHD1**	4	0.31
**FLG**	4	0.721
**FMN2**	4	0.0724
**PRUNE2**	4	0.113
**RYR2**	4	0.721
**SDK1**	4	0.548
**PCLO**	4	0.000191
**PDZD2**	4	0.756
**HMCN1**	4	0.136
**KMT2D**	4	0.939
**MUC16**	4	0.535
**PHF7**	4	0.79
**SMG1P7**	4	0.702
**ARID1A**	all	0.398
**WNK1**	all	0.32
**DNAH14**	all	0.592
**DNHD1**	all	0.0383
**FLG**	all	0.785
**FMN2**	all	0.501
**PRUNE2**	all	0.599
**RRS1**	all	0.958
**RYR2**	all	0.321
**SDK1**	all	0.199
**PCLO**	all	0.211
**PDZD2**	all	0.904
**HMCN1**	all	0.286
**KMT2D**	all	0.202
**MUC16**	all	0.322
**PHF7**	all	0.545
**SMG1P7**	all	0.324

Supplementary Table 6: UCSC database enrichment

stage	gene	PPI_enriched_gene	ratio
**1**	5	3	0.6
**2**	6	126	21
**3**	20	153	7.65
**4**	35	136	3.885714286

Supplementary Table 7: Edges weight larger than 3 after bootstrap

edgeList0	Stage1	Stage2	Stage3	Stage4
**VHL,PBRM1**	62	26	59	26
**VHL,TTN**	34	12	10	3
**VHL,MUC4**	30	8	48	44
**PBRM1,TTN**	19	4	4	0
**PBRM1,MUC4**	13	1	20	9
**VHL,MUC16**	12	0	3	0
**PBRM1,MUC16**	10	0	3	2
**VHL,RYR1**	8	0	0	0
**TTN,USH2A**	7	0	1	0
**VHL,KDM5C**	6	0	1	0
**PBRM1,SMG1P7**	6	0	5	0
**PBRM1,SETD2**	6	4	10	6
**MUC16,PRUNE2**	5	0	0	0
**VHL,SMG1P7**	5	2	2	0
**VHL,FMN2**	5	0	0	1
**VHL,SETD2**	5	2	7	11
**PBRM1,MUC6**	4	2	0	0
**FMN2,NFAT5**	4	0	0	0
**HSPA8,SPECC1**	4	0	0	0
**PBRM1,HSPA8**	4	0	0	0
**PBRM1,PDE4DIP**	4	0	0	0
**PBRM1,PRG4**	4	0	0	0
**VHL,PCNT**	4	0	0	0
**VHL,CSMD3**	4	0	1	0
**VHL,SSPO**	4	0	1	0
**PBRM1,LRP2**	4	0	2	0
**PBRM1,KDM5C**	4	0	1	4
**VHL,MTOR**	4	0	0	5
**PBRM1,VHL**	3	4	4	10
**MUC4,PBRM1**	2	1	6	10
**MUC4,TTN**	2	2	6	17
**VHL,HMCN1**	1	17	0	0
**MUC4,PCLO**	1	15	0	0

**VHL,RYR2**	1	14	0	0
**MUC4,FLG**	1	4	0	0
**VHL,KMT2C**	1	0	4	0
**VHL,KMT2D**	1	0	10	0
**MUC4,SMG1P7**	1	21	1	3
**PBRM1,SYNE2**	1	0	0	5
**VHL,DST**	1	0	0	5
**VHL,PDZD2**	1	0	0	10
**VHL,ARID1A**	0	31	0	0
**ARID1A,WNK1**	0	20	0	0
**BAP1,SMG1P7**	0	15	0	0
**SMG1P7,BAP1**	0	13	0	0
**SMG1P7,RRS1**	0	13	0	0
**HMCN1,SDK1**	0	10	0	0
**RYR2,DNHD1**	0	10	0	0
**VHL,WNK1**	0	10	0	0
**COL1A2,NOTCH2**	0	9	0	0
**PBRM1,MEGF6**	0	9	0	0
**SETD2,PDE4DIP**	0	9	0	0
**TTN,IGF2R**	0	9	0	0
**ADAMTS12,FCGBP**	0	8	0	0
**ARID1A,TRPC4**	0	8	0	0
**HMCN1,OBSL1**	0	8	0	0
**PBRM1,CDAN1**	0	8	0	0
**PBRM1,COL1A2**	0	8	0	0
**PDE4DIP,PRDM2**	0	8	0	0
**AHNAK2,DNAH9**	0	7	0	0
**MUC6,ZMYM2**	0	7	0	0
**SETD2,NCOA6**	0	7	0	0
**ARID1A,GOLGA5**	0	6	0	0
**COL1A2,RBM28**	0	6	0	0
**MUC4,MAST4**	0	6	0	0
**MUC6,EPPK1**	0	6	0	0
**NCOA6,PDE4DIP**	0	6	0	0
**PBRM1,ATP13A4**	0	6	0	0
**PCLO,ATP8A1**	0	6	0	0
**PCLO,MTMR11**	0	6	0	0
**SETD2,DMXL2**	0	6	0	0
**SETD2,MTOR**	0	6	0	0
**VHL,SDK1**	0	6	0	0
**BAP1,PREX1**	0	5	0	0
**BIRC6,AIDA**	0	5	0	0

**HMCN1,TTN**	0	5	0	0
**MUC4,GUCY1A3**	0	5	0	0
**MUC4,HRC**	0	5	0	0
**MUC4,RBM23**	0	5	0	0
**MUC6,CCDC74A**	0	5	0	0
**PBRM1,11-Sep**	0	5	0	0
**PCLO,SETD2**	0	5	0	0
**SETD2,CNTRL**	0	5	0	0
**TTN,NXPE2**	0	5	0	0
**WNK1,ARID1A**	0	5	0	0
**ABCC5,ATM**	0	4	0	0
**ADAMTS12,ODF2**	0	4	0	0
**COL1A2,CACNA1D**	0	4	0	0
**ITGA11,ZNF594**	0	4	0	0
**MUC4,GPR179**	0	4	0	0
**MUC4,KIAA2018**	0	4	0	0
**MUC4,PCK1**	0	4	0	0
**MUC6,MYLK3**	0	4	0	0
**MUC6,PER2**	0	4	0	0
**MUC6,VPS13A**	0	4	0	0
**NNT,EPHX1**	0	4	0	0
**NOTCH2,RBM28**	0	4	0	0
**PBRM1,COL5A3**	0	4	0	0
**PBRM1,GBF1**	0	4	0	0
**SETD2,PCLO**	0	4	0	0
**SETD2,PRDM2**	0	4	0	0
**TTN,ERCC1**	0	4	0	0
**TTN,RFTN1**	0	4	0	0
**TTN,TNRC6B**	0	4	0	0
**VHL,BDH2**	0	4	0	0
**VHL,CEP68**	0	4	0	0
**TTN,BRWD1**	0	7	1	0
**SMG1P7,NEFH**	0	6	1	0
**MUC4,VHL**	0	5	1	0
**MUC4,BAP1**	0	8	3	0
**MUC4,DNAH14**	0	0	4	0
**MUC4,FAT3**	0	0	4	0
**PBRM1,GLG1**	0	0	4	0
**PBRM1,RTTN**	0	0	4	0
**PKHD1L1,FOXD1**	0	0	4	0
**KMT2D,USH2A**	0	0	5	0
**MUC16,PTEN**	0	0	5	0

**MUC4,KMT2D**	0	0	5	0
**PBRM1,THSD7B**	0	0	5	0
**PBRM1,ZFHX4**	0	0	5	0
**VHL,BAP1**	0	5	6	0
**MUC4,USH2A**	0	0	6	0
**PBRM1,EGFR**	0	0	6	0
**PBRM1,KMT2C**	0	0	7	0
**PBRM1,SLC22A9**	0	0	7	0
**MUC4,MUC6**	0	4	0	1
**MUC4,SETD2**	0	2	4	2
**MTOR,SYNE2**	0	0	0	4
**PBRM1,FREM2**	0	0	0	4
**PBRM1,MTOR**	0	0	0	4
**TTN,DMBT1**	0	0	0	4
**VHL,FAM153A**	0	0	0	4
**DST,CROCC**	0	0	0	5
**PBRM1,SYNJ2**	0	0	0	5
**SYNE1,CACNA1C**	0	0	0	5
**SYNE1,KMT2C**	0	0	0	5
**SYNE2,UBR4**	0	0	0	5
**PBRM1,PHF7**	0	0	1	5
**VHL,DMKN**	0	0	1	5
**PBRM1,MED12L**	0	0	0	6
**PHF7,MUC5B**	0	0	0	6
**MUC4,DNMT1**	0	0	0	7
**MUC4,PHF7**	0	0	0	11
**MUC4,PRUNE2**	0	0	0	14
**VHL,PHF7**	0	0	0	19

Supplementary Fig 1: Logistic regression model of mutation and stage

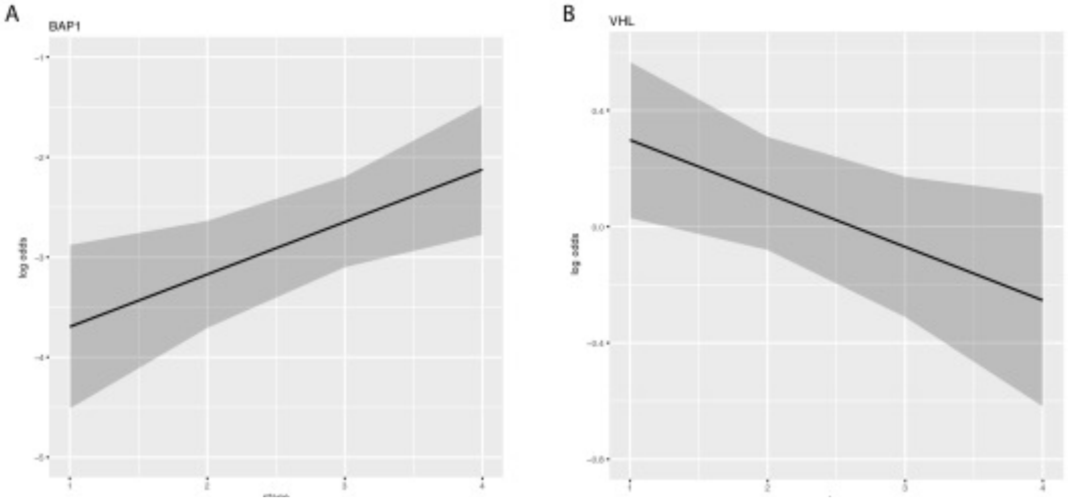

Supplementary Figure 2: Function enrichment of trunk genes. (A) GO slim. (B) GO database.

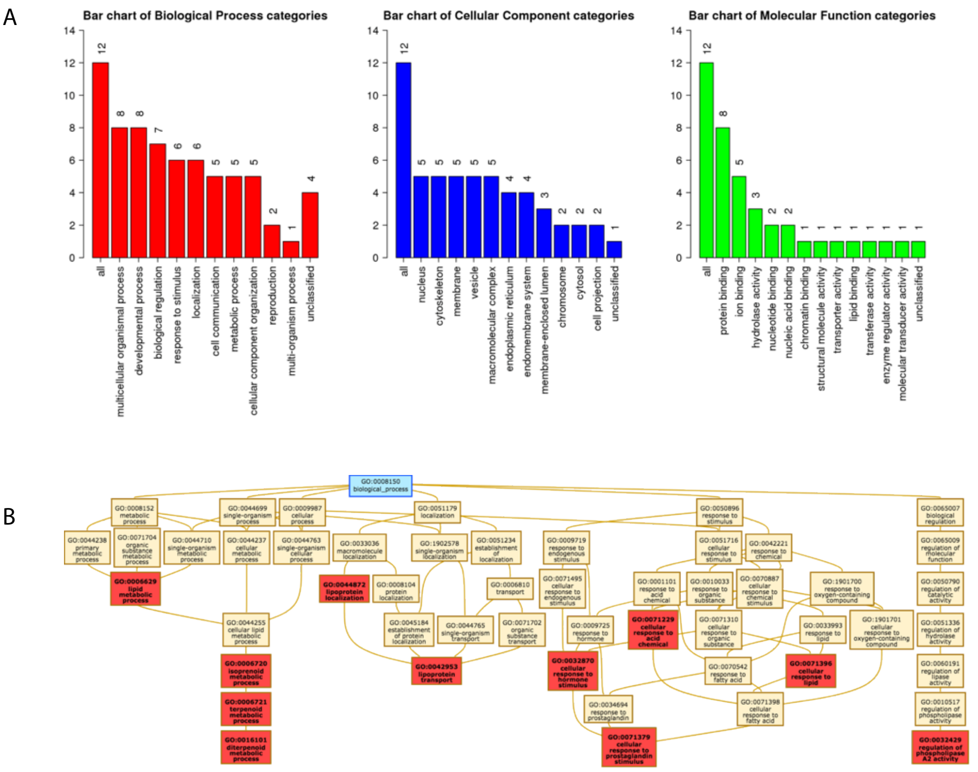

Supplementary Figure 3: Evolution DAG separate stages of raw data

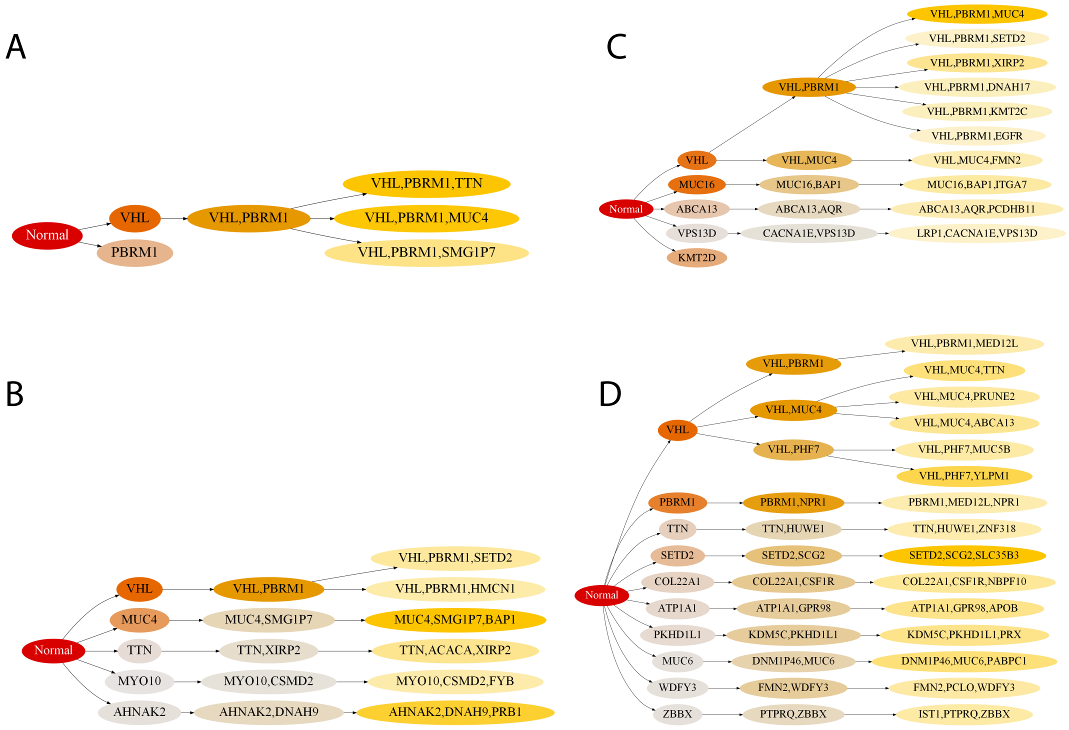

Supplementary Figure 4: Entropy distribution of high bootstrap score nodes and edges. (A) Nodes' entropy. (B) Edges' entropy. (C) Percentage of high bootstrap score nodes in total entropy. (D) Percentage of high bootstrap score edges in total entropy.

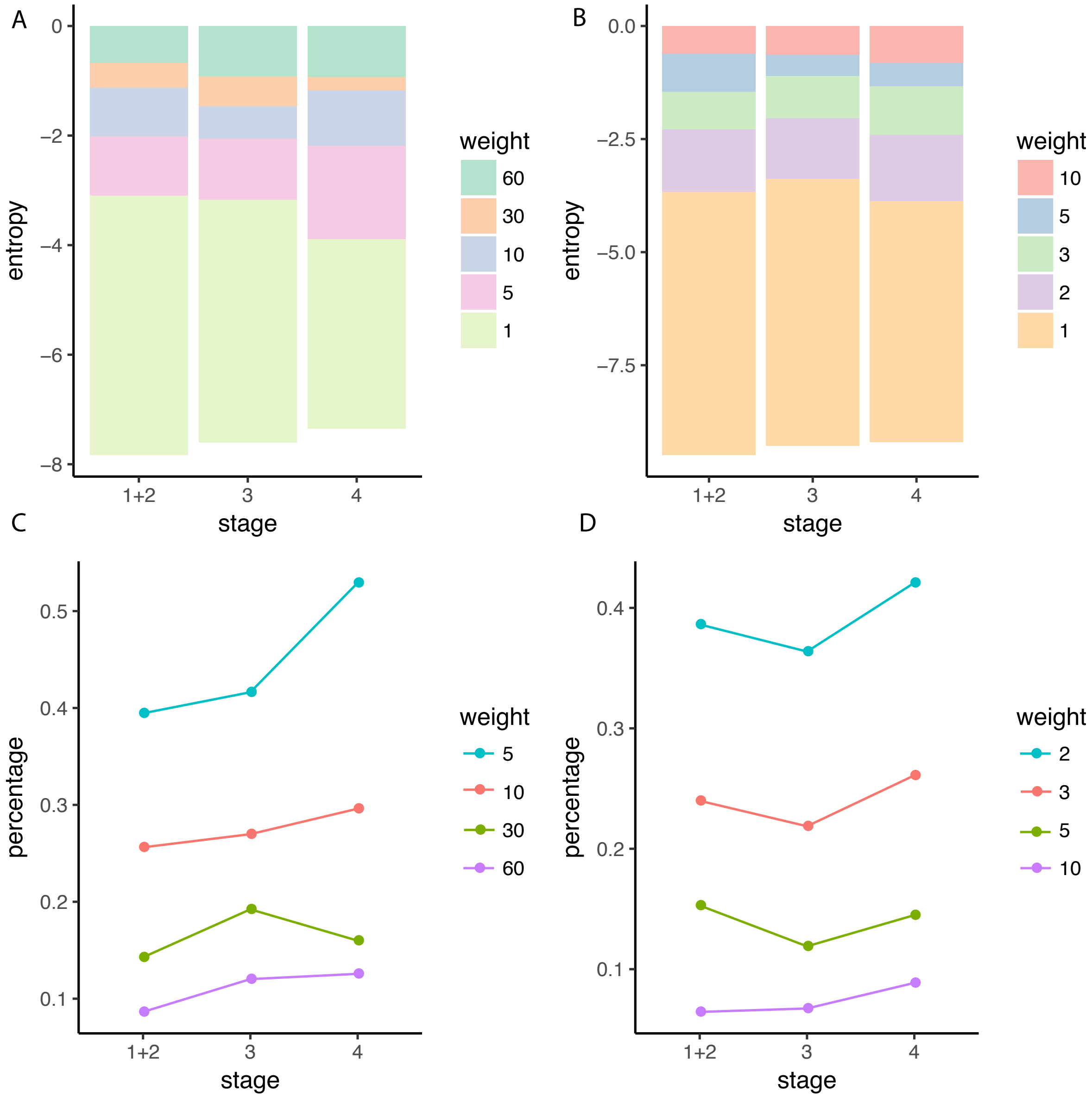

Supplementary Figure 5: PCLO stage IV expression survival curve (with low FMN2 expression)

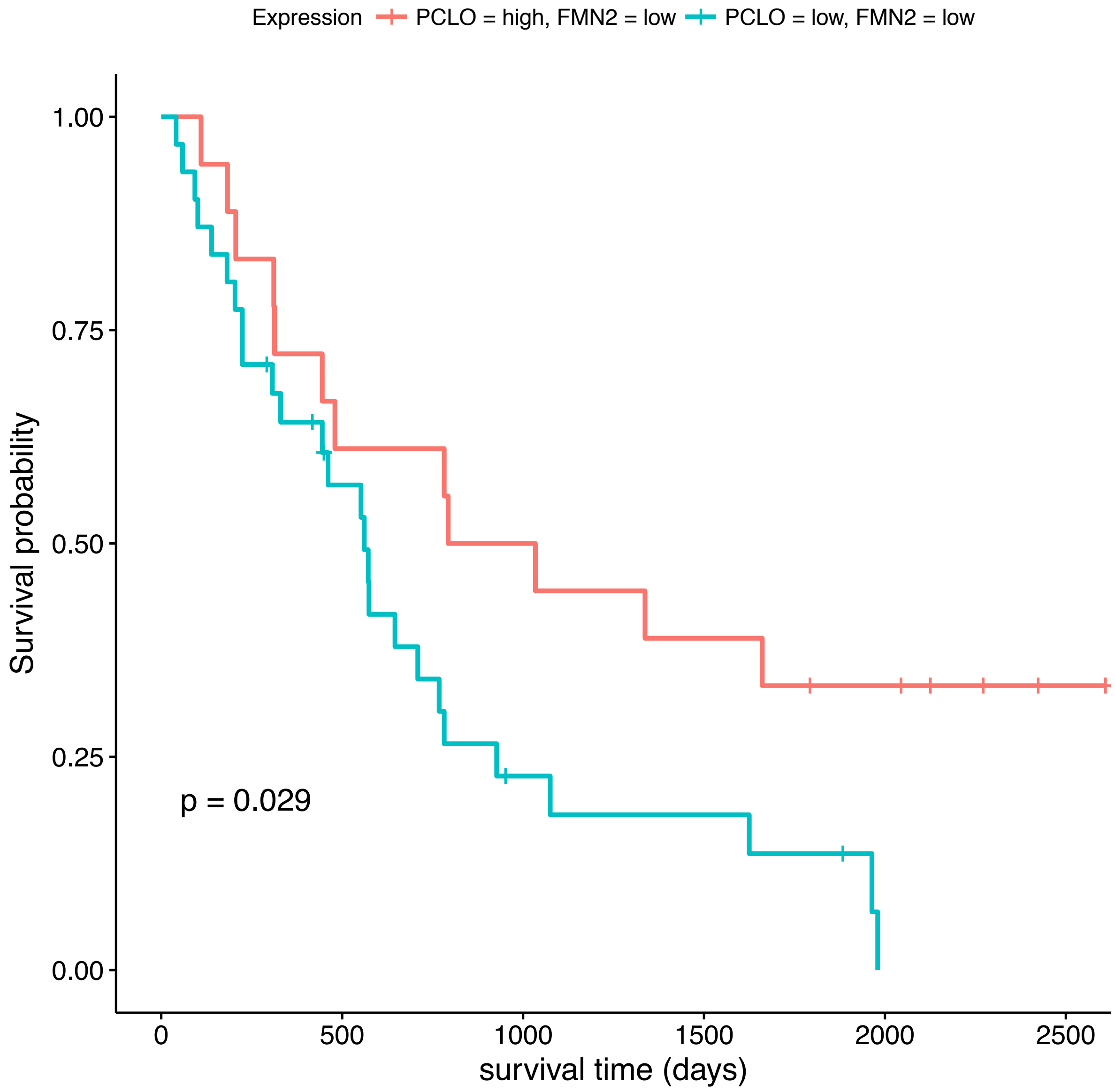

Supplement: Supplementary file 1 — Supplementary Information [file 41598_2018_20321_MOESM1_ESM.pdf]
